# Lipids as Regulators of Cellular Senescence

**DOI:** 10.3389/fphys.2022.796850

**Published:** 2022-03-04

**Authors:** Shruthi Hamsanathan, Aditi U. Gurkar

**Affiliations:** ^1^Aging Institute of UPMC, The University of Pittsburgh School of Medicine, Pittsburgh, PA, United States; ^2^Division of Geriatric Medicine, Department of Medicine, University of Pittsburgh School of Medicine, Pittsburgh, PA, United States; ^3^Geriatric Research, Education and Clinical Center, Veterans Affairs Pittsburgh Healthcare System, Pittsburgh, PA, United States

**Keywords:** cellular senescence, lipids, aging, bioactive lipids, senolytics

## Abstract

Lipids are key macromolecules that perform a multitude of biological functions ranging from maintaining structural integrity of membranes, energy storage, to signaling molecules. Unsurprisingly, variations in lipid composition and its levels can influence the functional and physiological state of the cell and its milieu. Cellular senescence is a permanent state of cell cycle arrest and is a hallmark of the aging process, as well as several age-related pathologies. Senescent cells are often characterized by alterations in morphology, metabolism, chromatin remodeling and exhibit a complex pro-inflammatory secretome (SASP). Recent studies have shown that the regulation of specific lipid species play a critical role in senescence. Indeed, some lipid species even contribute to the low-grade inflammation associated with SASP. Many protein regulators of senescence have been well characterized and are associated with lipid metabolism. However, the link between critical regulators of cellular senescence and senescence-associated lipid changes is yet to be elucidated. Here we systematically review the current knowledge on lipid metabolism and dynamics of cellular lipid content during senescence. We focus on the roles of major players of senescence in regulating lipid metabolism. Finally, we explore the future prospects of lipid research in senescence and its potential to be targeted as senotherapeutics.

## Introduction

Senescence is a cell-fate decision that is triggered in response to many different stressors including genotoxic stress, telomere attrition/damage, oncogene activation, and mitochondrial dysfunction (Herranz and Gil, 2018). In contrast to quiescence (cell cycle arrest), cellular senescence is a terminal state of growth arrest, where cells cannot re-enter the cell cycle despite mitogenic growth signals (Pack et al., 2019). Senescent cells resist apoptosis, at least partially due to an upregulation of survival pathways such as the BCL-2 family (Zhu et al., 2015; Yosef et al., 2016). Cellular senescence has been documented to have both beneficial and detrimental roles in maintaining health (He and Sharpless, 2017), and has been linked to embryonic development, wound healing, tumor suppression, as well as metastasis and aging. “Acute” senescence is a programmed cell-fate decision and is usually associated with tissue repair and homeostasis (Krizhanovsky et al., 2008; Jun and Lau, 2010; Rajagopalan and Long, 2012). In contrast, “chronic” senescence is associated with stochastic, persistent macromolecular damage and often associated with disease and aging (van Deursen, 2014). The permanent cell-cycle arrest of senescent cells is accompanied by a senescence associated secretory program (SASP). The SASP consists of pro-inflammatory factors such as cytokines, chemokines and extracellular matrix remodeling factors.

## Identification of Senescent Cells

**FIGURE 1:**
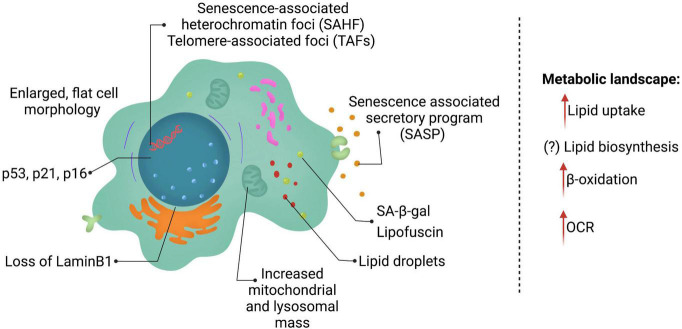
Features of senescent cells. Senescent cells are in a state of permanent cell cycle arrest and display several key features including lysosomal changes (SA-β-gal and lipofuscin), p53, p21, and/or p16 expression and a SASP program consisting of multiple cytokines, chemokines, and ECM remodeling enzymes. Senescent cells reprogram their metabolism as well heavily altering lipid metabolism.

The field is challenged by the lack of a single universal marker for cellular senescence. Currently, senescent cells are identified based on multiple markers (Figure 1), that allow for distinction between quiescent and senescent cells. The most prominent features of senescent cells include, an enlarged, flat cell morphology with an increase in the cytoplasm-to-nucleus ratio. A recent study suggested that the ratio of DNA to cytoplasm is critical to maintain cell function, and dysregulation of this ratio possibly contributes toward aging (Neurohr et al., 2019). Senescent cells also display an increase in the lysosomal enzyme, senescence-associated-β-galactosidase (SA-β-gal), and thus display increased SA-β-gal activity (Dimri et al., 1995; Hall et al., 2017). Single cell analysis, identified that cells with high SA-β-gal activity also exhibit larger cell size (Biran et al., 2017). It is important to note that SA-β-gal is often associated with senescent cells, but can be observed upon serum starvation or upon confluency in cell culture. Another feature of senescent cells is the accumulation of pigment granules in the lysosomes known as lipofuscin (Evangelou et al., 2017). However, lipofuscin accumulation is observed with age in many cells and organisms, including those that do not typically have other features of cellular senescence, e.g., *C. elegans* (Pincus et al., 2016). Lack of DNA replication and arrest of the cell cycle usually in G0/G1 phase are features of senescent cells, but do not distinguish between quiescence and senescence.

The two key signaling pathways upregulated for establishment and maintenance of senescence are p53/p21 and p16 (discussed in detail below). The burden of p21- and p16- positive expressing cells increases with age in mammals, however, currently the understanding of what percentage of cells are senescent is incomplete. A recent cross-sectional study in humans, examining three different age groups and multiple tissues, identified that p21- and p16-positive cells increase with age in the skin, pancreas and kidney (Idda et al., 2020). In contrast, p16-positive cells are observed in brain cortex, liver, spleen, and intestine (colon), whereas p21-positive cells increase in the dermis with age. Interestingly, some tissues, such as lung and muscle did not show any changes in these markers. Although a comprehensive study, it is important to note that this study was cross-sectional in design, as well as underlying health conditions may not have been fully examined. Nonetheless, a conclusion to be drawn from this study is that different cells and tissues accumulate different markers of senescence and may display varying burden of senescent cells.

Dynamic chromatin changes are a hallmark of senescent cells. Senescence-associated heterochromatin foci (SAHF) and telomere-associated foci (TAFs) are commonly used as markers for cellular senescence. However, these heterochromatin changes have been associated with senescence caused by nuclear DNA damage or telomere dysfunction, rather than other senescence-inducing stressors (Di Micco et al., 2011). A common chromatin feature in senescent cells is the generation of chromatin fragments that are released into the cytoplasm. The loss of nuclear lamina (Lamin B1) is observed in senescent cells, leading to “leaky” nucleus, and is thought to promote chromatin fragments (CCFs) in the cytoplasm (Di Micco et al., 2011; Dou et al., 2015, 2017). The CCFs activate the cyclic GMP–AMP synthase (cGAS) and the stimulator of interferon genes (STING) pathway (cGAS-STING pathway) and further establish SASP factors (Vizioli et al., 2020), thus reinforcing senescence and affecting neighboring cells.

Last but not least, an integral part of senescent cells is their ability to drive the SASP phenotype. SASP consist of pro-inflammatory cytokines [interleukin (IL)-1α, IL-1β, IL-6, and IL-8], chemokines (CCL2), and extracellular matrix remodeling factors such as matrix metalloproteinases (MMPs), serine/cysteine proteinase inhibitors (SERPINs) such as plasminogen activator inhibitor-1 (PAI-1) and tissue inhibitors of metalloproteinases (TIMPs) (Coppe et al., 2010; Eren et al., 2014). With advances in technology, recent studies have uncovered that different senescence-inducing stressors, as well as different cell types may lead to unique SASP being expressed (Basisty et al., 2020). SASP is thought to be transcriptionally controlled and released into the cytoplasm, as soluble factors or in exosomes (Lehmann et al., 2008; Ozcan et al., 2016; Takahashi et al., 2017), to influence neighboring cells (Ito et al., 2017). Understanding of SASP regulation is incomplete, although, p53, NF-κB, P38 MAPK, NOTCH, mTOR, and mitochondrial NAD+/NADH ratio have recently emerged as modulators of the SASP phenotype (Freund et al., 2011; Herranz et al., 2015; Nacarelli et al., 2019).

In this Review, we first describe the rewiring of lipid metabolism during cellular senescence. This review mostly focuses on cellular senescence observed with aging. We then review the link between major regulators of senescence and their role in lipid metabolism. Lastly, we discuss lipid classes that are altered in senescent cells and how they contribute to the senescence phenotype and outline tools that can help examine the causal role of lipids in driving cellular senescence.

## Metabolic Landscape of Senescent Cells

Aging is associated with dysregulated lipid metabolism (Mutlu et al., 2021). Several studies show that lipid composition changes with age in several species, including humans. Particularly, increased ratio of poly- and mono- unsaturated fatty acids have been recently found to be increased with age (Papsdorf and Brunet, 2019). However, whether cellular senescence, a key feature of aging is responsible for these lipid changes has been enigmatic so far. Lipids are an important fuel source that generate energy, as well as create crucial biological intermediates including lipid mediators, second messengers, and ketone bodies. Lipids can act as central metabolites that play a role in cellular signaling, both in an autocrine and paracrine manner, provide cell and organelle structure, and are fundamental for maintaining cellular homeostasis. Recent studies highlight the role of lipids in establishing cell fate decisions, such as cell division, apoptosis and cellular senescence (Ito and Ito, 2016). As described above, hallmarks of cellular senescence consists of increased mass of organelles such as, lysosomes and mitochondria, increased cellular size, loss of nuclear membrane integrity and SASP factors that can be “transported” by exosomes (Lizardo et al., 2017). Lipids are essential to each of these “senescence features,” including exosomes that are typically lipid enriched extracellular vesicles (Skotland et al., 2019) and therefore lipids are inextricably linked to the process of cellular senescence.

Several studies report an accumulation of lipid droplets in senescent cells compared to proliferating cells (Ogrodnik et al., 2014, 2019; Cox and Redman, 2017; Flor et al., 2017; Lizardo et al., 2017; Chee et al., 2021). Deregulated lipid accumulation in senescent cells can be due to increase in lipid uptake, upregulated lipid biosynthesis pathways or de-regulated lipid breakdown. Interestingly, senescent cells display increased lipid uptake (Flor et al., 2017), however, whether inhibition of lipid uptake can prevent or delays cellular senescence is unclear. Lipid levels are also controlled through a complex mechanism of lipid biosynthesis and breakdown. Advances in technology such as non-targeted “omic” approaches have highlighted the importance of lipid metabolism in cellular senescence. For example, a recent proteomic study of senescent cells, induced by DNA damaging agents, uncovered that lipid processing and metabolism genes including those involved in lipid binding, storage, biosynthesis and breakdown are severely altered (Flor et al., 2017). Fatty acids (FA) are essential building blocks for triglycerides (TGs) and phospholipids, and act as an energy source. Fatty acids are synthesized by the addition of malonyl-CoA to an acyl chain by one of two enzymes: acetyl-CoA carboxylase (ACCase) and fatty acid synthase (FAS). Although understudied, the role of FA biosynthesis in cellular senescence is possibly regulated in a context- and stress-dependent manner. Enlargement of cells and accumulation of membranous organelles such as, lysosomes and mitochondria are key hallmarks of senescence, and rely on lipid biosynthesis (Kim et al., 2010; Correia-Melo et al., 2016). Consistent with this theory, mouse hepatic stellate cells and human primary fibroblasts showed an increase in lipid biosynthesis, dependent on FASN, during initial establishment of cellular senescence (Fafian-Labora et al., 2019). FASN is upregulated prior to induction of late-stage senescence, and is increased with age in mouse liver. Interestingly, pharmacological or genetic inhibition of FASN, reduced p53-induced senescence, as well as, SASP (Borghesan et al., 2019; Fafian-Labora et al., 2019). Whether FASN is required for maintenance of cellular senescence and upregulated in a p53-independent manner is currently unknown. Oncogene-induced senescence (OIS) is a robust mechanism activated by oncogenic signaling due to an activating mutation of an oncogene or an inactivation of a tumor suppressor. Targeted metabolomic and bioenergetic analysis revealed that different modalities of senescence such as OIS and replicative senescence, led to very different metabolite patterns. In this study, Ras-induced OIS elevated levels of long chain free fatty acids (LCFA). Concomitantly, OIS reduced lipid biosynthesis by negatively regulating ACC, a rate limiting step for lipid biosynthesis (Quijano et al., 2012). It is important to note that these contradictory results about the role of lipid biosynthesis in senescence may be due to analyzing a different “stage” of cellular senescence, or different stress modality of senescence (aging versus OIS) or because of differences in cell-type (primary cells versus cancer cells).

Although the role of lipid biosynthesis in cellular senescence may not be straight-forward, FA breakdown has been consistently observed during senescence. A few reports indicate that lipid breakdown particularly through mitochondrial β-oxidation plays a paramount role in cellular senescence. Increased mitochondrial function has long been associated with senescence. Fatty acid oxidation (β-oxidation) is the mitochondrial multi-step process of breaking down a fatty acid into acetyl-CoA units. Long chain fatty acids (LCFA) are activated by acyl-carnitines and imported into the mitochondria for β-oxidation via a rate limiting enzyme carnitine palmitoyltransferase (CPT). Upon completion of β-oxidation, the generated acetyl-CoA can enter the TCA cycle, link to oxidative phosphorylation (OXPHOS) or can lead to acetylation of histones and other proteins. Indeed, recent data suggests that lipids contribute heavily toward histone acetylation and control gene transcriptional programs (McDonnell et al., 2016). During replicative senescence in myoblasts, several acyl-carnitines were increased, suggesting that senescent cells rely heavily on β-oxidation (Baraibar et al., 2016). Additionally, these senescent myoblasts had increased levels of monoacyl glycerols and glycerol-3-phosphate, as well as decreased mono- and polyunsaturated long chain fatty acids (>18 carbons), whereas medium chain fatty acids remained unaffected. This suggests that senescent cells increase lipid breakdown, however, whether this lipid mobilization and breakdown is used for energy production, gene transcription or generation of SASP (bioactive lipids) is unclear. Interestingly, an increase in FASN during establishment of the senescence program is also associated with an increase in CPT function and oxygen consumption rate (OCR). Consistent with this, inhibition of CPT1A during OIS, prevented cellular senescence and SASP establishment (Quijano et al., 2012). Collectively, these studies suggest that β-oxidation is required to drive cellular senescence (Fafian-Labora et al., 2019). It is important to note that one study reported that inhibition of β-oxidation could possibly promote senescence (Baraibar et al., 2016), Here, loss of CPT1C in lung fibroblasts, promoted lipid accumulation in cells and induced cellular senescence through lipotoxicity. One possibility is that lung fibroblasts uniquely depend on lipid β-oxidation, and inhibition of this process drives lipotoxicity and cellular senescence (Chen et al., 2021). Overall, these studies point toward major rewiring of lipid metabolism during establishment and maintenance of cellular senescence.

## Lipid Compositional Changes During Cellular Senescence

Although several studies show accumulation of lipid droplets (LDs) during cellular senescence, the content of these LDs may be far more critical. Lipids are divided into three groups, triglycerides (TAGs) (utilized for energy), steroids (cholesterol and hormones) and phospholipids (utilized for membrane structures). Here, we summarize the associated changes in composition of these lipids during senescence.

### Triglycerides

A study to decipher the transcriptomic and lipidomic changes during replicative senescence showed that lipid uptake mediated by CD36 is upregulated in senescent cells (Saitou et al., 2018). This feature is also consistent with DNA damage, oncogenic and chemical stress-induced senescence (Lizardo et al., 2017; Chong et al., 2018). Moreover, there is an enrichment of TAGs containing long- and very long-chain fatty acids, with at least one polyunsaturated fatty acyl (PUFA) chain (Saitou et al., 2018). Another study reported that DNA damage-induced senescence is accompanied with the upregulation of the triglyceride synthesis enzyme, diacylglycerol acyltransferase 1 (DGAT1). This study suggests that rather than lipid uptake, TAG biosynthesis may play a crucial role in establishment of senescence (Flor et al., 2017). The caveat though is that in this study the TAG species were not directly measured. It is therefore possible that both uptake and biosynthesis of TAGs may play a role in promoting cellular senescence.

### Cholesterol

Cholesterol homeostasis is critical to health. It is an important building block for hormones, vitamin D and integral to cell membrane integrity; however, excess cholesterol can have detrimental effects. Few studies have examined the role of cholesterol on cellular senescence. Naked mole rats (NMR) are known for their exceptional longevity, resilience to endogenous and exogenous stressors and cancer resistance. Interestingly, NMR fibroblasts had elevated levels of cholesterol-enriched lipid droplets (LDs), dependent on the Wnt/β-catenin pathway (Chee et al., 2021). Inhibition of cholesterol synthesis using lovastatin, an HMG-CoA reductase inhibitor, promoted senescence-like phenotype in NMR fibroblasts. Of note, only SA β-gal and 8-hydroxy-2′-deoxyguanosine (8-OHdG), a biomarker for DNA damaged by oxidative stress was measured in this study. Furthermore, the Wnt/β-catenin-cholesterol axis seems specific to NMR, as overexpression of β-catenin in mice or rat fibroblasts did not show any elevation in LDs (Chee et al., 2021). On the contrary, lipid profiling of OIS induced by ERBB2 (a member of the epidermal growth factor receptor), revealed increased cholesterol levels accumulated in vacuoles of senescent cells (Cadenas et al., 2012). This study suggests that high cholesterol levels are possibly a feature of senescence, although whether this accumulated cholesterol is a driver of senescence is not fully understood. In contrast, when mouse bone marrow stem cells (BMSCs) were exposed to low dose of cholesterol, it delayed oxidative stress-induced cellular senescence. Indeed, low levels of cholesterol promoted proliferation of BMSCs. Oxidative stress is known to damage macromolecular structures including cellular membranes, and since cholesterol is a major component of cell membranes, exogenous supplementation of cholesterol may help recovery from acute damage (Zhang et al., 2016). Further studies to examine the impact of cholesterol dosage on senescence *in vivo* is required to ascertain its exact role in the senescence phenotype.

### Phospholipids

Phospholipids are amphiphilic lipids that play important metabolic and structural roles. In a study looking at ERBB2 induced senescence (OIS), phospholipid with shortened acyl chains and unsaturated acyl chains in phosphatidylglycerol was observed (Cadenas et al., 2012), accompanied by increased membrane fluidity. This study also identified that mitochondrial lipids were altered, specifically, PG(34:1), PG(36:1) (increased) and LPE(18:1), PG(40:7) and PI(36:1) were reduced in senescent cells leading to loss of membrane potential. A comprehensive study compared and analyzed metabolites in replicative senescence, DNA damage-induced senescence and OIS in a human embryonic lung fibroblast (WI-38), using NMR spectroscopy. Interestingly, senescent cells displayed increased glycerophosphorylcholine-to-phosphocholine ratio. Importantly, glycerophosphorylcholine levels were particularly elevated in senescent cells compared to proliferating or quiescent cells, suggesting that this could be a unique feature of senescence (James et al., 2015). Bioactive phospholipids play an important role in “communication” and consist of lysophospholipids, ceramides, and sphingomyelin (Horn and Jaiswal, 2019). They can be released as secreted molecules or part of exosomes, and possibly play a role in extravesicular formation and release observed during senescence (Tepper et al., 2000; Nurminen et al., 2002; Trajkovic et al., 2008; Bianco et al., 2009; Hirsova et al., 2016). Hydrolysis of phospholipids by phospholipases, such as cPLA2, leads to lysophospholipids. An increase in lysophospholipids has been observed in senescent cells (Buratta et al., 2017; Narzt et al., 2021), and furthermore, lysophospholipids are shown to induce senescence in cholangiocytes (Shimizu et al., 2015). They can also act as a “find me” signal, but impair phagocytosis, possibly leading to inefficient clearance of senescent cells with age (Narzt et al., 2021). Phospholipids also influence the synthesis of eicosanoids that are known inflammatory mediators, thus contributing to the SASP phenotype (discussed in detail below). Thus, phospholipids play a varied role during senescence, including establishment of senescence, paracrine signaling and evasion of senescent cells.

## Major Lipid Players in Senescence

### Oxidized Fatty Acids

One of the major components of complex lipids in mammalian cells are polyunsaturated fatty acids (PUFAs) with atleast one bis allelic carbon and chain lengths ranging from 18 and above. PUFAs are precursors for potent metabolites that are involved in numerous pathophysiological events. One of the most well-studied families are eicosanoids, a class of oxygenated derivatives of arachidonic acid (C20) that include prostaglandins, leukotriens, lipoxins, hydroxy eicosatetraenoic acids, hydroperoxy eicosatetraenoic acids, and epoxy eicosatetraenoic acids. These bioactive lipids are derived enzymatically through the cyclooxygenase (COX), lipoxygenase (LOX), and cytochrome P450 (CYP) reactions or non-enzymatically through free radical reactions. Together, these bioactive lipids regulate diverse sets of both homeostatic and inflammatory processes. Pharmacological inhibition of the cysteinyl leukotrienes via LT antagonists, LY255283 and montelukast sodium, or ALOX5 inhibitor, BW-B70C, reduced the profibrotic effect of senescent cells on naive fibroblasts *in vitro* (Wiley et al., 2019) and attenuated TNFα-induced up-regulation of SA-β-Gal, p53, p21, and PAI-1 expression in primary human chondrocytes associated with osteoarthritis (Song et al., 2018). Increase in COX-2 activity has been shown to be associated with aging (Badawi et al., 2005; Chung et al., 1999; Baek et al., 2001). For example, COX-2 expression was upregulated in kidney, liver, heart and prostrate in old rodents (Hayek et al., 1997; Kim et al., 2001; Badawi et al., 2004; Choi et al., 2014; Tung et al., 2015), and in aging human tissues as well, especially kidney and skin (Melk et al., 2004; Kang et al., 2006; Surowiak et al., 2014). Although the cause- or-causal relationship is not clear, it was shown that in an inducible *Cox-2* transgenic mice, post-natal expression of COX-2 led to several premature aging phenotypes (Kim et al., 2016). Further, the lung fibroblasts from these animals expressed higher levels of senescence-associated SA-β-Gal, p16, p53, and phospho-H_2_Ax (Kim et al., 2016).

The age-related changes in COX-2 are often accompanied by an elevation in the prostaglandin synthesis. COX system is the major pathway catalyzing the conversion of arachidonic acid into prostaglandins, PGG2 and subsequently to PGH2 followed by the production of the bioactive lipids—PGE2, PGI2, PGD2, PGF2α—and thromboxane A2 by tissue-specific synthases. An upregulation in COX-2 expression and ensuing PGE2 production was observed during normal and stress-induced senescence of dermal, prostrate and lung human fibroblasts. Exogenous supplementation of arachidonic acid enhanced COX-2 activity and PGE2 production and subsequently accelerated the incidence of key senescence features like flat and enlarged cell morphology, SA-β-Gal activity and the cell cycle arrest (Dagouassat et al., 2013). In a study to understand the mechanisms of lung senescence linked with chronic obstructive pulmonary disease (COPD), it was shown that prostaglandin E2 released by pulmonary fibroblasts exerted both autocrine and paracrine effects to induce fibroblast senescence. This was accompanied with inflammation by EP2 and EP4 receptors, COX-2–dependent reactive oxygen species signaling (Dagouassat et al., 2013). Wiley et al. recently showed that senescent cells produced and accumulated 1a,1b-dihomo-15-deoxy-delta-12,14-prostaglandin J2, which together with other prostaglandin D2-related lipids promoted senescence arrest and SASP through activation of RAS signaling. In mice with drug-induced senescence, a dose of Navitoclax, a senolytic drug (eliminates senescent cells) elevated the levels of prostaglandin in blood and in urine. This study highlights the importance of prostaglandin lipids as biomarkers of senolysis.

Another primary oxidation product of arachidonic acid is hydroperoxy-eicosatetraenoic acid (hydroperoxyeicosatetraenoic acid, HpETE), generated via lipoxygenase (LOX) activity. Due to its regiospecificity nature, lipoxygenases are classified as arachidonate 5-, 8, 12-, and 15-lipoxygenase (5-LOX, 8-LOX, 12-LOX, and 15-LOX), which inserts oxygen at carbon 5, 8, 12, or 15 of arachidonic acid peroxidases. The primary oxidation products of HpETE are unstable and reduced further to its corresponding alcohol hydroxy-eicosatetraenoic acid (5-, 8-, 12-, and 15- hydroxy-6-trans-8,11,14-cis-eicosatetranoic acid, HETE) by glutathione peroxidase. The enzyme 5-LOX can act on 5-HETE to generate an unstable epoxide leukotriene A4 (LTA4), which can serve either as an intracellular intermediate in the synthesis of leukotriene B4/C4 (LTB4 and LTC4). Leukoterines (LTs) are the second major class of biologically active signaling lipids that plays a key role in inflammation and fibrosis. Idiopathic pulmonary fibrosis (IPF) is an interstitial lung disease with elevated senescent cell burden. It was shown that leukoterines secreted by senescent cells exacerbated IPF by promoting pulmonary fibrosis (Wiley et al., 2019) and kidney fibrosis by unilateral ureteral obstruction in mice (Kamata et al., 2019). Senescence induced irrespective of different modalities (oncogene-, stress-, and radiation-induced) in IMR-90 fibroblasts promoted secretion of leukoterines, as well as expression of enzymes involved in the biosynthesis of LTs (Wiley et al., 2019). Cysteinyl leukoterines such as LTC4 has been shown to accelerate acute kidney injury and drive renal pathogenesis in response to chemotherapy-induced senescence (Rubinstein and Dvash, 2018; Wiley, 2020). In line with these findings, leukotrienes increasingly released from senescent cells, irrespective of the senescence-inducer, and appear to function in parallel to SASP factors.

Besides lipoxygenases and cyclooxygenases, the third class of enzymes involved in eicosanoid synthesis are epoxygenases or cytochrome P450 (CYP) enzymes. Cytochrome P450 epoxygenases (CYP2C and CYP2J) synthesize epoxides, such as epoxyeicosatrienoic acid (EETs) in four different regioisomers (5, 6-, 8, 9-, 11, 12-, and 14,15-) that are recognized as specialized pro-resolving mediators (Alkayed et al., 1996; Zhang et al., 2007; Li et al., 2012). EETs have a wide range of protective actions such as anti-inflammatory, anti-apoptotic, vasodilatory and pro-angiogenic (Node et al., 1999; Iliff et al., 2009). The levels of EETs are often determined through production of dihydroxyeicosatrienoic acid (DHET) isoforms via soluble epoxide hydrolase (sEH) (Morisseau and Hammock, 2013). Gene deletion of soluble epoxide hydrolase was shown to improve blood flow and reduce infarct size following cerebral ischemia in reproductively senescent female mice (Zuloaga et al., 2014). 14,15-EET inhibited rat mesenteric arterial endothelial senescence, down-regulated p53 expression in aged endothelial cells and improved the impaired endothelium-dependent vasodilatation in aged rats through activation of mTORC2/Akt signaling pathway (Yang et al., 2014). Injection of 11,12-EET accelerated wound healing, tissue repair and reduced production of proinflammatory factors TNFα, IL-6, MMP and IL-1β in type 2 diabetic models of B6.VLepob/J (leptin-deficient, *ob/ob*) mice. In another study, both forms of EETs 11,12 and 14,15 epoxyeicosatrienoic acid rescued deteriorated wound healing in ischemic conditions (Sommer et al., 2019). Orally active subset of 14,15-EET analogs, termed EET-A, EET-B, and EET-C22 reduced cardiac and renal injury in spontaneous and angiotensin hypertension as well as promoted recovery after Ischemia/Reperfusion Injury by reducing contractile dysfunction (Campbell et al., 2017). Although several studies have highlighted the deleterious impact of cellular senescence in numerous chronic diseases, it is also recognized for its beneficial role in tumor suppression, wound healing and tissue regeneration. These studies highlight the contribution of CYP generated pro-resolvin lipid mediators in wound healing.

### Oxidized Phospholipids

Polyunsaturated fatty acyl containing phospholipids are prone to lipid peroxidation and form oxidized phospholipids (OxPLs) that play a significant role in apoptosis and inflammation. OxPLs are generated enzymatically through lipoxygenases or non-enzymatic attack through reactive oxygen species. However, irrespective of the mode, identical primary oxidation products (i.e., peroxyl radicals and hydroperoxides) are produced initially that subsequently undergo further oxidation in a stochastic manner and release a heterogenous mixture of bioactive OxPL species. In general, lipid peroxidation and levels of OxPLs increase with age. OxPLs generated via cytoplasmic hydroxyl radicals exacerbated cellular senescence in Toll-like receptor 4-knockout (TLR4-/-) OKD48- and IDOL-Tg mouse models (Sakai et al., 2019). Inhibition of TLR2 with oxid ized-1-palmitoyl-2-arachidonyl-sn-glycero-3-phosphorylcholine (OxPAPC), a well-studied oxidized product of a common phospholipid in mammalian cell membranes, 1-palmitoyl-2-arachidonoyl-sn-phosphatidylcholine (PAPC) significantly reduced IL-1α and IL-1β mRNA induction in oncogene induced senescence of IMR90 cells. In another study targeting OxPLs by transgenic overexpressing of E06-svFv, a single chain variable fragment that binds to phosphatidyl choline head group of OxPL attenuated age-associated trabecular bone loss in both female and male mice (Palmieri et al., 2021). Similar E06-svFv transgene mechanisms to inactivate OxPLs have been studied with age-associated diseases such as atheroscleosis, steatohepatitis (Sun et al., 2020) and macular degeneration (Handa et al., 2015) suggesting neutralizing OxPLs as a promising therapeutic target for some of the age-associated diseases. Cardiolipins, major phospholipids in mitochondria are extremely prone to lipid oxidation. With mitochondria being the critical organelle handling oxidative stress, oxidation of cardiolipin can lead to organelle dysfunction and generate lipid peroxides further exacerbating oxidative stress and promoting senescence. Knock out of ALCAT-1, an enzyme that promotes cardiolipin peroxidation protected against onset of cellular senescence and preserved mitochondrial function by reducing sensitivity to lipid oxidation.

Greenberg et al. (2008) proposed the “lipid whisker” hypothesis as a novel feature of membrane architecture in senescent or apoptotic cells. These cells are distinguished by the presence of protruding “whiskers” as a result of phospholipid oxidation via lipid peroxidation or oxidative stress. The oxPL whiskers such as oxidized phosphatidylserine contribute to pattern recognition for common receptors like CD36 that play a major role in engulfment and phagocytosis of senescent cells (Podrez et al., 2002; Greenberg et al., 2006, 2008).

### Sphingolipids

Sphingolipids are a class of complex phospholipids with a hydrophobic core consisting of an amino alcohol, sphingosine and a long-chain fatty acid chain. Bioactive forms this class such as sphingosine, sphingosine-1-phosphate, ceramide, ceramide-1-phosphate, dihydroceramide are known to modulate a plethora of cellular functions such as cell migration, growth regulation, adhesion, apoptosis, senescence and inflammatory responses (Hannun and Obeid, 2018).

Several studies have highlighted the role of sphingolipids in both development and aging (Giusto et al., 1992; Lightle et al., 2000; Cutler and Mattson, 2001; Hannun and Obeid, 2018). For example, aging induces accumulation of sphingosine and ceramides in liver and brain tissues and alterations in sphingolipid metabolism increases the risk and progression of age-related disease (Giusto et al., 1992; Lightle et al., 2000). A systemic study on sphingolipid metabolic enzymes in kidney, liver and brain tissues of day-1 to 720-day-old rats revealed a significant increase in activities of the sphingolipid catabolic enzymes (SMase and ceramidases) (Sacket et al., 2009). Impairment of ceramide and sphingolipid synthesis genes in yeast, worms and flies extended lifespan (Cutler and Mattson, 2001; Rao et al., 2007; Yi et al., 2016).

In senescent human dermal fibroblasts (HDF), ceramide levels and a sphingomyelinase activity were markedly elevated. Exogenous ceramide induced senescent phenotype in young HDF at concentrations that mimics endogenous ceramide levels in senescent cells and recapitulated molecular changes of senescence, suggesting ceramides act as mediators of senescence (Venable et al., 1995). Of note, in therapy-induced senescence, exogenous addition of sphingomyelin increased ceramide levels that induced apoptosis and decreased senescence. This is possibly explained by ceramide levels- no inhibition of cell cycle progression at low ceramide levels, induction of senescence at moderate levels, and initiation of apoptosis at high levels (Modrak et al., 2009). In another study, deficiencies in ceramide transfer protein (CERT) in primary mouse embryonic fibroblasts led to senescence and dysregulated sphingolipid metabolism by increasing hexosylceramides production (Rao et al., 2007). Increased hexoceramides such as glucosylceramide led to age-related impairments in CD4(+) T cell function and immunosenescence (Molano et al., 2012). Several studies have suggested that sphingolipid metabolism as effectors or modulators of the p53 tumor suppressor pathway, particularly sphingomyelinase and sphingosine kinase I (SK1), which are directly regulated by p53, contributing to ceramide-induced senescence in cells (Dbaibo et al., 1998; Oskouian et al., 2006; Heffernan-Stroud and Obeid, 2011). For example, the expression of sphingosine kinase I (SK1), a downstream target of p53, was significantly elevated in p53-knockout mice that developed thymic lymphoma, and p53-deficient mice lacking SK1 were protected from thymic lymphoma. Mechanistic studies revealed that p53 tumor suppression caused by loss of SK1 was mediated by increased sphingosine and ceramides, resulting in tumor senescence. Non-canonical sphingolipids such as deoxyceramides has been shown to be depleted during tumor-induced senescence and increasing the levels of 1-deoxyceramides reduced the senescence phenotype in human colorectal cancer cells. The imbalance in deoxyceramide levels can affect sphingolipid signaling and alter membrane remodeling (Millner et al., 2020). Despite the fact that several evidences point to the multiple roles of bioactive sphingolipids in aging, these intriguing findings warrant more research into the functional role of sphingolipids in senescence.

### Lysophospholipids

Lysophospholipids are multifunctional lipid mediators and are formed from hydrolyzed phospholipids with either an alkyl or acyl chain (Rivera and Chun, 2008). The lysophospholipids (LPLs) are broadly categorized as lysoglycerophospholipids and lysosphingolipids. The common ones in circulation are lysophosphatidylcholine (LPC), lysophosphatidic acid (LPA), lysophosphatidylserine (LPS), sphingosine, sphingosine-1 phosphate (S1P), and sphingosylphosphorylcholine (SPC) that are derived from corresponding phospholipids. Among the LPLs, lysophosphatidylcholine is the most abundant class (Ojala et al., 2007) and activates multiple signaling pathways that are involved in oxidative stress and inflammatory responses (Kakisaka et al., 2012; Bansal et al., 2016). They are also major secretory components of extracellular vesicles, which has recently gained a lot of attention as key players within the secretome of senescent cells (Urbanelli et al., 2016; Buratta et al., 2017). For example, biochemical characterization of extracellular vesicles released by fibroblasts subjected to H-Ras induced senescence indicated a significant enrichment in LPCs species (16:0; 16:1; 18:1; 18:2; 20:4, and 26:0). Both, replicative- and stress- induced-senescent dermal fibroblasts showed elevated levels of lysophosphatidylcholines. The LPC exhibited SASP activity by eliciting chemokine release in non-senescent fibroblasts and obstructed TLR2–6/CD36 signaling in macrophages (Narzt et al., 2021). Cholangiocyte senescence in biliary tract cancer has been linked to an increase in LPC levels and the resulting cytotoxicity. LPCs induced upregulation of components of SASP interleukin-8 (IL-8), IL-6, transforming growth factor-β and PAI-1, as well as p21 and SA-β-gal activity in MMNK-1 human cholangiocyte cells.

**FIGURE 2:**
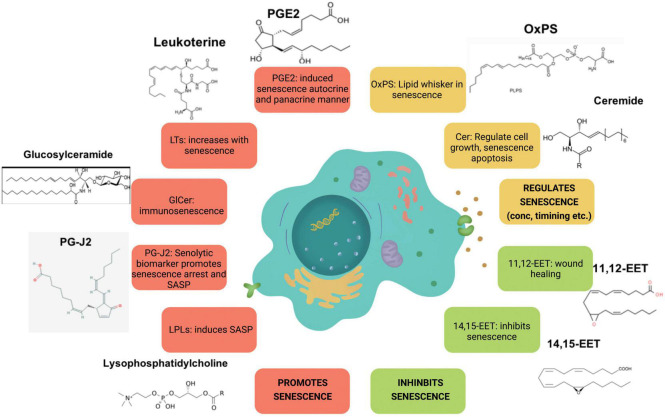
Major lipid species involved in senescence. Species illustrated in red increase with senescence, whereas species in orange and green regulate and inhibit it, respectively.

Studies have also shown association between aging and lysophosphatidic acid (LPA) signaling. For instance, alterations in LPA1 receptor was linked to the presence of depression and cognitive deficits in the elderly population (Moreno-Fernandez et al., 2018). Age-dependent changes in cAMP profiles were induced by LPA as noted in young and senescent human fibroblasts (Jang et al., 2006). LPA were detected at significantly higher concentrations in the cerebral cortex synaptosomes of aged rats (Pasquare et al., 2009). Knockdown of LPA3 led to cell senescence in mesenchymal stromal cells (Kanehira et al., 2017). Another finding showed that LPA regulates ROS levels and cell senescence through LPA3 to alleviate cell aging in Hutchinson–Gilford progeria syndrome (Chen et al., 2020). Together these studies reveal a key role of lysophospholipid signaling and its wide spread effects in the process of aging and senescence. Figure 2 illustrates the key lipid players involved in senescence.

## Major Molecular Players of Cellular Senescence and Their Role in Lipid Regulation

### p16

**FIGURE 3:**
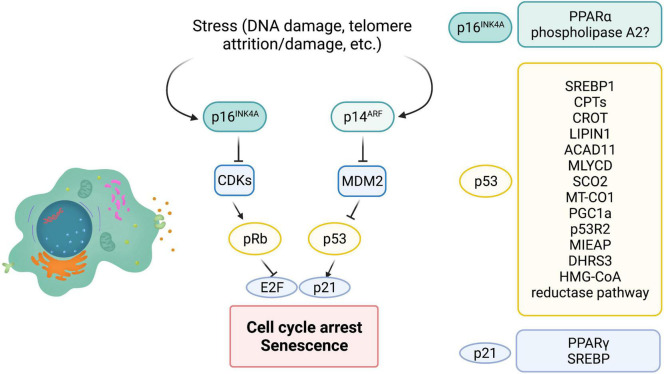
The role of major players of senescence in lipid metabolism. P16^*INK*4*a*^, p53, and p21 are major cell cycle regulators that control cellular senescence. INK4a/ARF encodes both p16 and ARF. MDM2 is an E3 ligase that ubiquitinates and leads to degradation of cellular senescence. ARF can inhibit MDM2 and therefore stabilize p53. P21 is a bonafide target of p53 and is partially responsible for cell cycle arrest and cellular senescence. P16 inhibits cyclin dependent kinases (CDK) thus maintaining Rb in a hypophosphorylated state and preventing E2F transcription factor activity. On the right are modulators of lipid metabolism that are regulated by p16, p53, and p21 (? = Possibly regulated by p16).

Cellular senescence is initiated and maintained through two critical pathways: p16^*INK*4*A*^/Rb and/or p53/p21^*cip*1^. It is important to note that both, p16 and p53, are well known tumor suppressors. p16 inhibits cyclin dependent kinases, CDK4 and CDK6, thus maintaining Rb in a hypophosphorylated form and blocking cell cycle progression (Figure 3). Expression of p16 is a key factor for maintaining long-term senescence (Serrano et al., 1997; Yamakoshi et al., 2009). A recent study isolated high expressing p16-positive cells, specifically macrophages, and showed that these cells exhibit several other markers of senescence, including SA-β-gal, reduced proliferation and accompanying SASP (Liu et al., 2019). This suggests that high expression of p16 is indeed associated with cellular senescence. Additionally, several single nucleotide polymorphisms (SNPs) have been identified in the *CDKN2a/b* locus (encoding the *p16INK4a*, *ARF*, and *p15INK4b* transcripts) and associated with multiple age-related pathologies (Jeck et al., 2012; Fortney et al., 2015). Furthermore, p16 expression is largely undetectable in young mice, Krishnamurthy et al. (2004), Liu et al. (2009) but is strongly associated with aging in multiple mouse tissues (Burd et al., 2013). Similarly, other “drivers” that accelerate aging features, for example high fat diet, drives p16 expression through epigenetic modulation of its coding region (Zhang et al., 2018). Consistent with p16 being a major player in cellular senescence, genetic ablation of p16-positive cells, using the INK-ATTAC (Baker et al., 2011) and p16-3MR mice (Demaria et al., 2014), reduced senescent cell burden and improved healthspan, both in a progeria model, as well as naturally aged WT animals. Of note, preventing the accumulation of p19*^ARF^*-positive cells, another tumor suppressor that regulates p53 stability did not delay or prevent age-related pathologies (Baker et al., 2008), suggesting that p16 is crucial for maintenance of senescence, at least in mice models.

In humans, peripheral blood T lymphocytes exhibit increased expression of p16^*INK*4*a*^ with age, tobacco smoking and physical inactivity. Similarly, p16-positive senescent T cells increase with age in mice (Yousefzadeh et al., 2021). In addition to aging, senescent T cells have been isolated from a number of tumors including lung and breast cancer (Meloni et al., 2006; Gruber et al., 2008; Urbaniak-Kujda et al., 2009). These senescent T cells from the tumor microenvironment seem to be dysfunctional and have reduced antitumor activity (Liu et al., 2018; Zhao et al., 2020). Interestingly, such senescent T cells with increased p16 expression display unbalanced lipid metabolism, leading to accumulation of lipid droplets (Liu et al., 2021), consistent with senescent cells in other tissues. Inhibition of the group IVA phospholipase A_2_, reprogrammed metabolism and prevented T cell senescence, suggesting that unbalanced lipid metabolism could be a key driver of T cell senescence (Liu et al., 2021). However, whether p16 directly controls lipid metabolism is still unknown in this model. One recent study did identify a direct link between p16 expression and lipid metabolism. During fasting, inhibition of p16 in primary hepatocytes enhanced activation of PPARα, a transcription factor with a major role in lipid metabolism, and increased fatty acid oxidation (Deleye et al., 2020; Figure 3). Consistent with this, p16 overexpression reduced mitochondrial activity and fatty acid oxidation, and led to accumulation of lipid droplets both *in vitro* and *in vivo*. However, p16 is known to have roles outside of senescence and these studies did not analyze cellular senescence in this fasting model. Of note, several studies (described above) suggest that elevated lipid β-oxidation is observed in senescent cells. Therefore, further studies exploring if p16 controls lipid catabolism during senescence will uncover insights into how p16 maintains long-term senescence.

### p53

p53 is one of the most well-studied tumor suppressor, and is activated in response to various stressors including DNA damage, telomere attrition, mitochondrial, oxidative or oncogenic stress. Post-translational modification of p53, including phosphorylation, acetylation, sumoylation, ubiquitination and neddylation, regulate its activity. Upon acute stress, p53 is activated and can modulate DNA repair and quiescence (Vousden and Prives, 2009; Kasteri et al., 2018). However, chronic activation of p53 promotes cellular senescence (Sharpless and Depinho, 2006; Salama et al., 2014). p53 indirectly downregulates the expression of several genes required for cell cycle progression. Although the role of p53 in regulating the cell cycle is well-understood, recent studies indicate that p53 can also regulate metabolism and in particular lipid metabolism.

p53 enhances fatty acid oxidation and oxidative phosphorylation (OXPHOS), while inhibiting fatty acid synthesis. The role of p53 in suppressing lipid synthesis is prominent in the p53 knock out mouse models that exhibit obesity (Wang S. J. et al., 2016). Interestingly, induction of cellular senescence increases oxygen consumption and inhibits fatty acid synthesis, suggesting that p53 may play a pivotal role in rewiring the metabolic circuitry of senescent cells (Quijano et al., 2012). Inhibition of fatty acid synthesis by p53 is predominantly through repression of the master regulator of fatty acid synthesis, *SREBP1* (Yahagi et al., 2003). SREBPs control the expression of multiple lipogenic enzymes, including *acetyl-CoA carboxylase* (*ACC*), *ATP citrate lyase* (*ACLY*), and *fatty acid synthase* (*FASN*) (Yahagi et al., 2003; Eberle et al., 2004). These observations stem from a model of obese (*ob/ob*) mice, that displayed elevated *p53* and a concomitant downregulation of SREBP-1 and its targets, possibly by reducing promoter activity. Indeed, *p53* deletion in the *ob/ob* mice partially rescues SREBP-1 expression (Yahagi et al., 2003).

Carnitine palmitoyltransferase 1A and 1C (CPT1A, CPT1C), the rate limiting enzyme for fatty acid oxidation and aid in the transport of fatty acids into the mitochondria are transcriptionally regulated by p53 (Sanchez-Macedo et al., 2013). Carnitine *O*-octanoyltransferase (CROT) another enzyme that facilitates export of fatty acids from the peroxisomes has also been identified as a p53 target (Goldstein et al., 2012). Once exported from the peroxisomes these fatty acids can undergo mitochondrial β-oxidation. Similarly, Acad11 that catalyzes the first step of mitochondrial fatty acid oxidation, is a transcriptional target of p53 (Jiang et al., 2015). DNA damage with doxorubicin, an agent that induces double strand breaks and activates p53, upregulated Acad11 expression in a p53-dependent manner (He et al., 2011; Jiang et al., 2015). Malonyl-CoA decarboxylase (MLYCD) is a mitochondrial enzyme that converts malonyl-CoA to acetyl-CoA and a critical regulator of fatty acid oxidation. In a recent study in mice exhibiting ribosomal stress, MLYCD transcription was controlled through p53 binding to its promoter and intron regions, and reduced lipid accumulation in the liver (Liu et al., 2014). Another bonafide p53 target, Lipin1 has three p53 binding sites in intron 1, and is important for adipose tissue development. DNA damage, particularly double strand breaks (DSBs) by γ-irradiation, that activates p53 leads to increased Lipin1 expression in several tissues including spleen and thymus (Assaily et al., 2011). Recent data in mouse myoblast C2C12 cells, shows that upon glucose starvation p53 upregulates Lipin 1 and fatty acid oxidation (Assaily et al., 2011). Additionally, Lipin1 has also been shown to interact with PGC-1α, another p53 target, to promote fatty acid oxidation (Finck et al., 2006; Sen et al., 2011). Clearly, activated wild-type p53 does seem to play a role in lipid metabolism, particularly fatty acid oxidation. However, whether chronic p53 activation that is known to promote cellular senescence, leads to similar changes has not yet been fully elucidated. Fatty acid oxidation is linked to OXPHOS, and provides reduced equivalents to the OXPHOS pathway. p53 has several targets that play a role in OXPHOS, including *cytochrome c oxidase 2* (*SCO2*) (Matoba et al., 2006) and the *mitochondrial encoded cytochrome c oxidase 1* (*MT-CO1*) (Okamura et al., 1999). p53 is also shown to translocate into the mitochondria and can interact with mitochondrial DNA polymerase γ (Achanta et al., 2005), control mitochondrial biogenesis through PGC1α, regulate mitochondrial DNA copy number and mitochondrial mass through *p53-controlled ribonucleotide reductase* (*p53R2*) (Bourdon et al., 2007) and is also involved in clearance of dysfunctional mitochondria by mitophagy through mitochondria-eating protein (Mieap) (Kitamura et al., 2011).

Interestingly, short-chain dehydrogenase reductase (DHRS3) that is localized to lipid droplets has also been identified as a target of p53 (Beilstein et al., 2016). There are two p53 responsible elements in the *DHRS3* promoter, and several reports show that activated p53 promotes buildup of lipid droplets concomitant with an increase in DHRS3 (Kirschner et al., 2010; Deisenroth et al., 2011). In addition to FAO, OXPHOS and lipid droplet dynamics, p53 is also involved in cholesterol homeostasis. Caveolin 1 is a structural protein that binds to cholesterol. In an *in vitro* study, in human dermal fibroblasts, *caveolin 1* was induced upon overexpression of p53 (Bist et al., 2000). This led to reduced intracellular cholesterol, suggesting the possible role of p53 in cholesterol efflux. The HMG-CoA reductase pathway is important for cholesterol synthesis and several enzymes in this pathway are p53 targets including 3′-hydroxy-3′-methylglutaryl-coenzyme A reductase, MVA kinase, farnesyl diphosphate synthase and farnesyl diphosphate farnesyl transferase 1 (Laezza et al., 2015). However, there are opposing reports on whether p53 activates or represses the HMG-CoA reductase pathway. Both studies use WT p53 expressing cell lines, however, one study uses astrocytes and glioblastoma cell lines whereas the other uses mouse embryonic fibroblasts, SK-HEP-1 and HCT116 cells (Laezza et al., 2015; Moon et al., 2019; Figure 3). Currently the role of HMG-CoA reductase pathway in cellular senescence is not clear, although a few reports suggest that inhibiting this pathway in pre-malignant cells promotes cellular senescence and inhibits tumorigenesis.

### p21

A bonafide transcriptional target of p53 is p21^*WAF*1/CIP1^, a 21 kDa protein encoded by the CDKN1A gene (el-Deiry et al., 1993). p21 interacts with cyclin dependent kinases (CDKs), thus maintaining RB in a hypophosphorylated state and inhibiting cell cycle progression (Harper et al., 1993; Chen et al., 1996). Since, p21 binds several cyclin/CDK complexes, including CDK1, 2, 4 and 6, it can promote arrest at any stage of the cell cycle. In contrast p16 specifically binds to inactivate CDK4 and CDK6, thus arresting cells in G0/G1 phase (Harper et al., 1993; Pavletich, 1999). p21 can also bind proliferating cell nuclear antigen (PCNA) (Waga et al., 1994) and inhibit the cell cycle. It is important to note that p21 can be activated in a p53-independent manner (Aliouat-Denis et al., 2005; Abbas and Dutta, 2009). As mentioned above, p16 expression is required to maintain a senescent state. In contrast, p21 is crucial for initiation of senescence (Noda et al., 1994; Hernandez-Segura et al., 2017). Indeed, mice lacking p21^*WAF*1/CIP1^ showed defects in embryonic senescence leading to developmental defects (Munoz-Espin et al., 2013; Storer et al., 2013). Of note, embryonic senescence is a transient program thought to be beneficial and is resolved in a timely manner. Furthermore, several reports show that p21 expression does not persist in senescent cells (Stein et al., 1999; Sharpless and Sherr, 2015).

Role of p21 in lipid metabolism is not well understood. p21 does have an apparent role in adipocyte maintenance, however, outside of adipogenic effects not much is known about p21 in regulating lipid metabolism. There are some studies that report p21 as a pro-adipogenic factor. Knockdown or loss of p21 inhibited adipocyte differentiation (Inoue et al., 2008). Consistent with this, phosphorylation of p21 by MPK-38 led to nuclear translocation and increased association with peroxisome proliferator-activated receptor γ (PPARγ), thus inhibiting the role of PPARγ in adipogenesis. Furthermore, in this study expression of p21 rescued the metabolic phenotypes observed. Contradicting these studies, loss of p21 showed an increase in adipocyte hyperplasia (Naaz et al., 2004). In this study loss of p21 led to spontaneous adipose conversion and adipocyte hyperplasia in mice. However, effects in the study of Inoue et al. were observed in the terminal differentiation phase of the adipogenic program, whereas the anti-adipogenic effects were evident in the earlier determination phase of adipogenesis. Interestingly, p21 was identified as a target of SREBPs. Both SREBP-1a and SREBP-2 binding sites exist in the p21 promoter (Inoue et al., 2005). Furthermore, SREBP-1a led to p21 expression and cell cycle inhibition, under conditions of lipid deprivation. Consistent with this observation, SREBP null mice had reduced expression of p21 (Figure 3). It will be of great interest to examine whether expression of p21 leads to differential lipid changes during “acute” versus “chronic” senescence. Although, p16, p53, and p21 are key molecular players in cellular senescence their exact role in modulating lipid metabolism, reprogramming lipid composition and generation of bioactive lipid species is not well explored.

## Potential Role of Lipids as Therapeutics in Targeting Senescence

Cellular senescence is a defining feature of aging, as well as a contributor to chronic non-healing wounds and general tissue aging pathology. Several key bioactive lipids such as cytochrome P450 (CYP)-derived epoxyeicosatrienoic acids (EETs), leukoterines, lipoxins described above are known to play a significant role in tissue repair. In fact, EETs have been shown as a viable angiogenic therapeutic strategy for its ability to accelerate wound epithelization (Sander et al., 2013). Long term inhibition of PGE2 degradation significantly reduced pulmonary fibrosis lesions in mouse model of bleomycin-induced lung senescence. In contrast, profibrotic leukotriene synthesis is elevated in IPF. Although PGE2 inhibition holds great promise as a stand-alone treatment or in combination with currently approved therapies for idiopathic pulmonary fibrosis, modulating eicosanoid metabolism in context of senescence can open up therapeutic avenues and provide mechanistic clues into tissue aging. Although the above studies have highlighted the beneficial role of senescent cells in wound healing, it is still a paradox as to how these senescent cells also impair wound healing in aging. A temporal lipidome analysis of senescent cells from different tissues would provide insights into lipid changes that occur in senescent cells of aged tissues, as well as allow for the development of tailored senescence-targeting therapeutics.

## Tools to Identify Functional Lipids and Current Challenges

The ubiquitous and structurally diverse nature of lipids molecules and its varied abundance across cells and tissues contributes to the complexity in the process of lipid identification. There are various techniques available for studying individual lipids and the lipidome, each with its own set of constraints. Understanding the benefits and limitations of these approaches is therefore crucial in identifying lipids within a biological model. With the emergence of lipidomics, there has been several advancements in analytical techniques such as soft ionization methods, high resolution mass spectrometry, tandem mass spectrometry, MS imaging and development of spectral libraries and lipid database such as LIPIDMAPS. This has led to not only identifying novel lipids but also unravel mechanisms responsible for pathophysiological conditions, biomarkers for early diagnosis and drug targeting (Quehenberger et al., 2010; Wang M. et al., 2016). Advances in high-resolution lipidomics platforms have made it possible to investigate lipidome changes associated with aging and age-associated diseases (Wong et al., 2019). For example, both targeted and untargeted lipidomics approaches provided insights into phospholipid remodeling in senescent cells, led to the identification of 15d-PGJ2 as a biomarker of senolysis and elevated ceramide C16:0 levels as prognostic marker for functional decline (Wennberg et al., 2018; Millner and Atilla-Gokcumen, 2020; Wiley et al., 2021). Despite advancements made in this field, the complexity in terms of chemistry, diversity, and abundances necessitates the continued pursuit of technical innovation. For example, identifying certain isomers of eicosanoids, a variety of sphingolipids, phospholipid intermediates are still challenging to be quantified accurately. Developing the informatics tools or libraries to interpret a multitude of lipid species and large sets of lipidomics data has been challenging. Nevertheless, a lot of progress has been made toward fine tuning of high-resolution mass spectrometry and precise identification of lipid structures. Multi-omic data integration approaches, dynamic lipidomics to asses turnover kinetics, single cell lipidomics has generated a lot of interest and holds promise in advancing the field forward.

## Unanswered Questions in the Field

With advances in technology, we have unraveled that lipids do play a role in cellular senescence. However, we are left with several unanswered and interesting questions: Do different stages of cellular senescence (e.g., initiation versus late senescence) have different lipid compositions? If so is it causal in initiation or maintenance of senescence? While the lipids discussed in this review have been linked to senescence, more research is needed to establish cause or consequence effect. In addition to this, the link between major senescent players- p53, p21, or p16, and lipid alterations is still to be uncovered. Importantly, it will be interesting to understand whether lipids could act as biomarkers for senescence and/or senolysis, hence making intervention possible in the near future.

Cellular senescence, in summary is a cell-fate process associated with several age-related pathologies including frailty, Alzheimer’s disease, Parkinson disease, atherosclerosis, idiopathic pulmonary fibrosis, chronic obstructive pulmonary disease, and osteoarthritis (Schafer et al., 2017; Chinta et al., 2018; Sagiv et al., 2018). Indeed, the National Institutes of Health (NIH) has recently started a new initiative, The Common Fund’s Cellular Senescence Network (SenNet) Program, to better understand the key hallmarks of cellular senescence and map the burden of senescent cells in human. This further highlights the critical need of understanding cellular senescence. Additionally, directed removal of senescent cells or associated SASP improves health considerably, at least in animal models (Zhu et al., 2015; Jeon et al., 2017; Bussian et al., 2018; Guerrero et al., 2019; Zhang et al., 2019). Further understanding the role of lipids in cellular senescence will open new therapeutic approaches for several age-related diseases.

## Author Contributions

SH and AUG reviewed the literature, authored the review, contributed to the article, and approved the submitted version.

## Conflict of Interest

The authors declare that the research was conducted in the absence of any commercial or financial relationships that could be construed as a potential conflict of interest.

## Publisher’s Note

All claims expressed in this article are solely those of the authors and do not necessarily represent those of their affiliated organizations, or those of the publisher, the editors and the reviewers. Any product that may be evaluated in this article, or claim that may be made by its manufacturer, is not guaranteed or endorsed by the publisher.
